# Association of Hospital Resource Utilization With Neurodevelopmental Outcomes in Neonates With Hypoxic-Ischemic Encephalopathy

**DOI:** 10.1001/jamanetworkopen.2023.3770

**Published:** 2023-03-21

**Authors:** Vilmaris Quinones Cardona, Rakesh Rao, Isabella Zaniletti, Priscilla Joe, Yvette R. Johnson, Robert DiGeronimo, Shannon E. Hamrick, Kyong-Soon Lee, Ulrike Mietzsch, Girija Natarajan, Eric S. Peeples, Tai-Wei Wu, Tanzeema Hossain, John Flibotte, Amit Chandel, Amy Distler, Jeffrey S. Shenberger, Onome Oghifobibi, An N. Massaro, Maria L. V. Dizon

**Affiliations:** 1 St Christopher’s Hospital for Children, Drexel University College of Medicine, Philadelphia, Pennsylvania; 2 St Louis Children’s Hospital, St Louis, Missouri; 3 Children’s Hospital Association, Kansas City, Kansas; 4 UCSF Benioff Children’s Hospital, Oakland, California; 5 Cook’s Children’s Medical Center, Department of Pediatrics, Texas Christian University Medical School, Fort Worth; 6 Seattle Children’s Hospital, University of Washington, Seattle; 7 Children’s Healthcare of Atlanta and Emory University, Atlanta, Georgia; 8 The Hospital for Sick Children, Toronto, Ontario, Canada; 9 Children’s Hospital of Michigan, Central Michigan University, Detroit; 10 University of Nebraska Medical Center, Omaha; 11 Children’s Hospital of Los Angeles, USC Keck School of Medicine, Los Angeles, California; 12 Boston Children’s Hospital, Boston, Massachusetts; 13 Children’s Hospital of Philadelphia, Philadelphia, Pennsylvania; 14 Atrium Health Wake Forest Baptist, Wake Forest School of Medicine, Winston-Salem, North Carolina; 15 University of Pittsburg Medical Center, Pittsburg, Pennsylvania; 16 Childrens National Health Systems, Washington, DC; 17 Ann & Robert H. Lurie Children’s Hospital of Chicago, Northwestern University Feinberg School of Medicine, Chicago, Illinois

## Abstract

**Importance:**

Intercenter variation exists in the management of hypoxic-ischemic encephalopathy (HIE). It is unclear whether increased resource utilization translates into improved neurodevelopmental outcomes.

**Objective:**

To determine if higher resource utilization during the first 4 days of age, quantified by hospital costs, is associated with survival without neurodevelopmental impairment (NDI) among infants with HIE.

**Design, Setting, and Participants:**

Retrospective cohort analysis of neonates with HIE who underwent therapeutic hypothermia (TH) at US children’s hospitals participating in the Children’s Hospitals Neonatal Database between 2010 and 2016. Data were analyzed from December 2021 to December 2022.

**Exposures:**

Infants who survived to 4 days of age and had neurodevelopmental outcomes assessed at greater than 11 months of age were divided into 2 groups: (1) death or NDI and (2) survived without NDI. Resource utilization was defined as costs of hospitalization including neonatal neurocritical care (NNCC). Data were linked with Pediatric Health Information Systems to quantify standardized costs by terciles.

**Main Outcomes and Measures:**

The main outcome was death or NDI. Characteristics, outcomes, hospitalization, and NNCC costs were compared.

**Results:**

Among the 381 patients who were included, median (IQR) gestational age was 39 (38-40) weeks; maternal race included 79 (20.7%) Black mothers, 237 (62.2%) White mothers, and 58 (15.2%) mothers with other race; 80 (21%) died, 64 (17%) survived with NDI (combined death or NDI group: 144 patients [38%]), and 237 (62%) survived without NDI. The combined death or NDI group had a higher rate of infants with Apgar score at 10 minutes less than or equal to 5 (65.3% [94 of 144] vs 39.7% [94 of 237]; *P* < .001) and a lower rate of infants with mild or moderate HIE (36.1% [52 of 144] vs 82.3% [195 of 237]; *P* < .001) compared with the survived without NDI group. Compared with low-cost centers, there was no association between high– or medium–hospitalization cost centers and death or NDI. High– and medium–EEG cost centers had lower odds of death or NDI compared with low-cost centers (high vs low: OR, 0.30 [95% CI, 0.16-0.57]; medium vs low: OR, 0.29 [95% CI, 0.13-0.62]). High– and medium–laboratory cost centers had higher odds of death or NDI compared with low-cost centers (high vs low: OR, 2.35 [95% CI, 1.19-4.66]; medium vs low: OR, 1.93 [95% CI, 1.07-3.47]). High–antiseizure medication cost centers had higher odds of death or NDI compared with low-cost centers (high vs. low: OR, 3.72 [95% CI, 1.51-9.18]; medium vs low: OR, 1.56 [95% CI, 0.71-3.42]).

**Conclusions and Relevance:**

Hospitalization costs during the first 4 days of age in neonates with HIE treated with TH were not associated with neurodevelopmental outcomes. Higher EEG costs were associated with lower odds of death or NDI yet higher laboratory and antiseizure medication costs were not. These findings serve as first steps toward identifying aspects of NNCC that are associated with outcomes.

## Key Points

**Question:**

Is higher resource utilization, quantified by hospital costs, associated with survival without neurodevelopmental impairment among infants with hypoxic-ischemic encephalopathy treated with therapeutic hypothermia?

**Findings:**

In this cohort study of 381 neonates with hypoxic-ischemic encephalopathy who underwent therapeutic hypothermia in 11 children’s hospitals, total hospitalization costs during the first 4 days were not associated with neurodevelopmental outcomes. Higher electroencephalography (EEG) costs were associated with survival without neurodevelopmental impairment, yet higher antiseizure medication or laboratory costs were not.

**Meaning:**

These findings suggest EEG monitoring may improve outcomes, whereas more intensive laboratory monitoring may not yield similar benefits.

## Teaser

This cohort study examines whether there is an association between higher resource utilization during the first 4 days of age, quantified by hospital costs, and survival without neurodevelopmental impairment among infants with hypoxic-ischemic encephalopathy (HIE).

## Introduction

Therapeutic hypothermia (TH) is, to date, the most effective therapy to improve neurodevelopmental outcome in neonates with hypoxic-ischemic encephalopathy (HIE).^1^ Although TH is a cost-effective management strategy, practices and costs vary widely across centers, as reported previously.^2^

Laboratory monitoring, electroencephalography (EEG), and antiseizure medications (ASM), which are central to contemporary neonatal neurocritical care (NNCC) during the first 4 days of admission for HIE, are variably used among centers.^3,4,5,6^ At some sites, laboratory testing is highly protocolized throughout treatment, whereas others allow testing based on clinician discretion. EEG use may vary by center depending on availability of full montage video EEG (vEEG) or amplitude integrated EEG (aEEG), and the presence of technicians and electrophysiologists. Similarly, we previously reported significant intercenter variation with regards to ASM use in HIE.^7^ Utilization of ASM may vary based on center guidelines (ie, criteria to initiate and/or discontinue ASM), use of continuous EEG to identify electrographic seizures, and/or individual clinician practice.

Although there is wide intercenter variation with regards to resource utilization in HIE,^2,8,9^ it remains unclear whether increased (or decreased) resource use is associated with improved long-term outcomes. We sought to determine if higher resource utilization, measured by standardized costs during the first 4 days of age in the neonatal intensive care unit (NICU), a period corresponding to TH and rewarming, is associated with survival without neurodevelopmental impairment (NDI) among infants with perinatal HIE. A secondary objective was to determine if specific NNCC costs (ie, laboratory, EEG, and ASM costs) are associated with outcomes.

## Methods

This is an analysis of linked data from 3 multicenter data sources described later from neonates with HIE treated at 11 regional US NICUs participating in the Children’s Hospitals Neonatal Consortium. Cost was used as an indirect measure of resource utilization during the first 4 days of age for HIE admission and for ascertaining use of specific NNCC components such as laboratory, EEG, and ASM. This study follows the Strengthening the Reporting of Observational Studies in Epidemiology (STROBE) reporting guideline. The institutional review board (IRB) at each participating institution approved participation in the Children’s Hospitals Neonatal Database (CHND) and additional IRB approval was obtained at centers participating in this study to collect developmental follow-up data. Waiver of consent was granted by the IRBs at the 11 participating centers as this was a minimal risk retrospective database study.

### Data Sources

The CHND prospectively captures detailed clinical data from all infants admitted to participating level IV NICUs.^8^ Data regarding antenatal, maternal, birth, and delivery characteristics and clinical data from the NICU stay, including neurological data regarding HIE presenting characteristics, presence of clinical or electrographic seizures, and neuroimaging findings, were abstracted according to the CHND manual of operations.^8^ CHND provides descriptive data that is largely categorical but does not quantify frequency or duration of NNCC services. The Pediatric Health Information Systems (PHIS) contains detailed hospital administrative and billing data from more than 40 pediatric institutions.^2^ Methods ensuring data quality for both databases have been reported.^2,8,10,11^ Developmental outcome data were retrospectively collected at individual participating sites.^12^ CHND, PHIS, and developmental outcome data were linked at the patient level using unique identifiers unavailable to investigators.

### Study Population

The CHND^8^ was queried to identify neonates born at participating centers between July 2010 and July 2016 with the diagnosis of perinatal HIE according to established criteria,^5^ treated with TH, admitted less than 2 days of age, at least 36 weeks’ gestation and 1800 g at birth. Neonates were excluded if they had major congenital anomalies, if linkage to PHIS was not possible, or if they did not survive through the first 4 days of age. Survivors were excluded if neurodevelopmental assessments were not available at greater than 11 months of age. Maternal race and ethnicity data were collected as reported from the CHND; these data were collected to assess if these factors were associated with outcome. Race and ethnicity groups included were White, Black, or other (Asian, American Indian or Alaska Native, Native Hawaiian or Other Pacific Islander).

### Estimation of Resource Utilization

PHIS Clinical Transaction Classification (CTC) codes corresponding to hospital billing for admission and any type of laboratory test, EEG, and ASM charge were used to quantify standardized costs as a reflection of frequency and duration of resource use. Standardized costs were calculated according to a previously described cost master index.^2,13^ Briefly, costs for every CTC billing code were computed based on a ratio of cost to charge and adjusted for geographical region wage and price index. A standardized unit cost for each CTC code was defined as the median of unit costs across all PHIS-participating hospitals and tabulated in a cost master index (CMI) as previously described.^2,13^ The CMI-based standardized cost was used to estimate hospitalization costs for every admission in the study cohort. Total cost of hospitalization was calculated per patient and per center for the first 4 days, to allow for evaluation of patient- and/or clinician-level and center-level variation as potential risk factors of outcome. Analogous costs were also calculated for laboratory tests, EEG, and ASMs during this study period.

### Calculation of Developmental Index

Because all sites in the consortium did not use the same developmental assessments, we defined NDI at greater than 11 months of age if the participant had any of the following: substantial developmental delay (defined based on the specific assessment tool used), diagnosis of cerebral palsy (defined by documentation of the diagnosis by a neurologist or developmental specialist and/or a Gross Motor Function Classification Scale (GMFCS) score ≥2), or presence of deafness (requiring amplification) or blindness. For participants assessed using the Bayley Scales of Infant Development, Third Edition (BSID-III), NDI was defined by a BSID-III composite cognitive score less than 85 or a composite motor score less than 70.^14^ For participants assessed using the Capute Scales or the Ireton Infant Development Inventory, NDI was defined as a developmental quotient less than 70.^15,16,17^ These definitions are similar to moderate or severe disability used in HIE cooling trials.^18^

### Statistical Analysis

Study sample size was based on a convenience sample of consecutive admissions of infants meeting inclusion criteria during the study period. Study population characteristics and cost distribution data were described using standard summary statistics. Cost data were log transformed to account for the skewed distribution. Costs were categorized as low, medium, and high based on thresholds derived from calculating terciles at both the individual case and center levels. Univariable and multivariable logistic regression analyses were performed to assess the associations between costs and outcome groups. Analyses were adjusted for covariates including HIE severity, EEG confirmed seizures, inhaled nitric oxide (iNO), extracorporeal membrane oxygenation (ECMO), ventilator days and socioeconomic status (SES; reflected by median household income within the maternal postal zip code, based on US census statistics). To evaluate the association between center EEG and ASM costs, the 25th and 75th percentile of the center median EEG costs were plotted on the vertical axis and of the median center ASM costs on the horizontal axis. The significance level was set at 2-sided *P* < .05. Data were analyzed from December 2021 to December 2022 using SAS Enterprise Guide version 7.1 (SAS Institute).^19^

## Results

**Figure 1.**
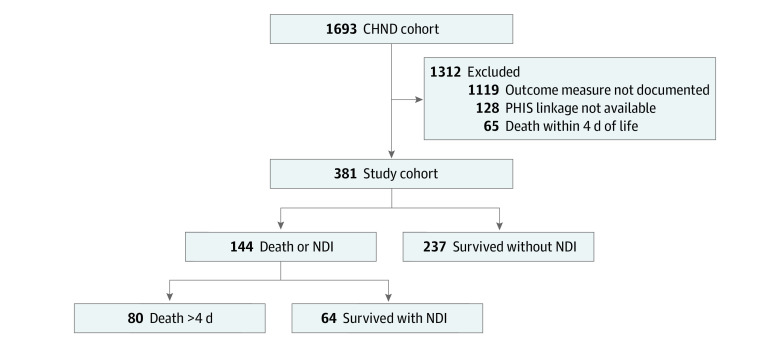
Study Population CHND indicates Children’s Hospitals Neonatal Database; NDI, neurodevelopmental impairment; PHIS, Pediatric Health Information System.

A total of 1693 patients with HIE were entered into the CHND during the study period. Of the 574 patients that had outcome measures, PHIS linkage was unavailable for 128 patients and 65 patients died within the first 4 days of age. The final analysis included 381 patients; of these, 80 (21%) died, 64 (17%) survived with NDI, and 237 (62%) survived without NDI (Figure 1); median (IQR) gestational age was 39 (38-40) weeks; maternal race included 79 (20.7%) Black mothers, 237 (62.2%) White mothers, and 58 (15.2%) mothers with other race. Characteristics of all infants excluded are described in eTable 1 in Supplement 1. Characteristics of infants excluded due to death within the first 4 days of age are listed in eTable 2 in Supplement 1. Characteristics of those lost to follow-up are listed in eTable 3 in Supplement 1.

### Clinical Characteristics

Infants who died or survived with NDI compared with those who survived without NDI were more likely to have Apgar score less than or equal to 5 at 10 minutes (65.3% [94 of 144] vs 39.7% [94 of 237]; *P* < .001), be born at home (3.5% [5 of 144] vs 0.8% [2 of 237]; *P* < .001) and less likely to have mild to moderate HIE (36.1% [52 of 144] vs 82.3% [195 of 237]; *P* < .001) (Table 1). Rates of NDI components are provided in eTable 4 in Supplement 1.

**Table 1.** Study Population Characteristics and Outcomes

Characteristics	No. (%)			*P* value
	Overall (n = 381)	Death >4 d or NDI (n = 144)	Survivors without NDI (n = 237)	
Gestational age, median (IQR), wk	39 (38-40)	39 (38-40)	39 (38-40)	.40
Birth weight, median (IQR), g	3314 (2950-3788)	3364 (2940-3819)	3280 (2960-3751)	.57
Sex				
Female	162 (42.5)	62 (43.1)	100 (42.2)	.92
Male	219 (57.48)	82 (56.94)	137 (57.81)	.91
Maternal race				
Black	79 (20.7)	34 (23.6)	45 (19.0)	.30
White	237 (62.2)	84 (58.3)	153 (64.6)	.23
Other	58 (15.2)	21 (14.6)	37 (15.6)	.88
Unknown	7 (1.8)	5 (3.5)	2 (0.8)	.11
Maternal ethnicity				
Hispanic	63 (16.5)	24 (16.7)	39 (16.5)	.99
Birth location				
Birthing center, not hospital	2 (0.5)	2 (1.39)	0 (0)	NA
Home	7 (1.8)	5 (3.5)	2 (0.8)	<.001
Hospital	372 (97.6)	140 (97.2)	232 (97.9)	.72
Delivery type				
Vaginal, nonoperative	107 (28.1)	35 (24.3)	72 (30.4)	.24
Vaginal, operative	37 (9.7)	17 (11.8)	20 (8.4)	.29
Cesarean	237 (62.2)	92 (63.9)	145 (61.2)	.66
APGAR at 10 min ≤5	188 (49.3)	94 (65.3)	94 (39.7)	<.001
Presenting pH <7	82 (21.5)	33 (22.2)	50 (21.1)	.79
Cord gas BD (or first hr gas), median (IQR)	16.4 (11-21)	18 (14-23)	15 (10-20)	.04
Perinatal sentinel event				
Nuchal cord	72 (18.9)	29 (20.1)	43 (18.1)	.69
Cord prolapse	15 (3.9)	6 (4.2)	9 (3.8)	.99
Uterine rupture	24 (6.3)	24 (9.7)	10 (4.2)	.05
Placental abruption	48 (12.6)	18 (12.5)	30 (12.7)	.05
Fetal distress	80 (21.0)	39 (27.1)	41 (17.3)	.03
Encephalopathy severity				
Mild moderate	247 (64.8)	52 (36.1)	195 (82.3)	<.001
Severe	116 (30.5)	86 (59.7)	30 (12.7)	.81
Unknown	18 (4.7)	6 (4.2)	12 (5.1)	<.001
Head cooling	59 (15.5)^a^	23 (15.9)	36 (15.2)	.88
Whole body cooling	326 (85.6)^a^	125 (86.8)	201 (84.8)	.65
Clinical seizures	51 (13.4)	19 (13.2)	32 (13.5)	.99
EEG/aEEG confirmed seizures	105 (27.6)	57 (39.6)	48 (20.3)	<.001
No seizures	202 (53.0)	57 (39.6)	145 (61.2)	<.001
ECMO	12 (3.2)	4 (4.2)	6 (2.5)	.38
iNO	74 (19.4)	40 (27.7)	34 (14.3)	.005
Tracheostomy	2 (0.5)	0 (0)	2 (0.8)	NA
Gastrostomy tube	20 (5.3)	9 (6.3)	11 (4.6)	.49
Ventilator time, median (IQR), d	4 (0-7)	6 (4-9)	1 (0-5)	<.001
Length of stay, median (IQR), d	12 (8-20)	10 (7-19)	13 (9-21)	.002

Abbreviations: aEEG, amplitude integrated electroencephalogram; BD, base deficit; EEG, electroencephalography; ECMO, extracorporeal membrane oxygenation; iNO, inhaled nitric oxide; NA, not applicable; NDI, neurodevelopmental impairment.

^a^ The sum of head and whole-body cooling is greater than our n = 381 as some infants were noted to receive both.

### Short-term Outcomes

Infants in the death or NDI group were more likely to have electrographically confirmed seizures (39.6% [57 of 144] vs 20.3% [48 of 237]; *P* < .001), received iNO (27.7% [40 of 144] vs 14.3% [34 of 237]; *P* = .005), and had higher median (IQR) ventilator time (6 [4-9] days vs 1 [0-5] days; *P* < .001) than infants who survived without NDI. The death or NDI group had a shorter median LOS (10 [7-19] days vs 13 [9-21] days; *P* < .001). The need for ECMO did not differ between groups (Table 1).

### Unadjusted Costs

Costs and comparisons between groups are presented in Table 2. Unadjusted median hospitalization costs in the first 4 days were higher for the death or NDI group compared with survived without NDI ($29 482 [20 964-34 030] vs $25 284 [20 964-34 030]; *P* = .001). Among NNCC costs, laboratory costs were highest, accounting for 14% of total costs, followed by EEG, accounting for 9% of total costs. Laboratory costs and ASM costs were higher in the death or NDI group. EEG costs were higher in the survived without NDI group (Table 2). Unadjusted costs further stratified by outcomes of death, survived with NDI and survived without NDI, show highest hospitalization costs in death after 4 days (eTable 5 in Supplement 1) and lowest in death within 4 days (eTable 6 in Supplement 1).

**Table 2.** Unadjusted Costs per Case for First 4 Days of Hospitalization in US Dollars

Costs	Median (IQR), $			*P* value
	Total	Death >4 d or NDI (n = 144)	Survivors without NDI (n = 237)	
Hospitalization, first 4 d	26 420 (21 664-36 610)	29 482 (20 964-34 030)	25 285 (20 964-34 030)	.001
EEG	2417 (1293-4567)	1861 (1260-4197)	2454 (1462-4644)	.09
Laboratory	3695 (1984-5425)	4624 (3424-6933)	2707 (1570-4698)	<.001
Antiseizure medications	40 (17-100)	55 (185-149)	34 (16-81)	.02

Abbreviations: EEG, electroencephalography; NDI, neurodevelopmental impairment.

### Adjusted Costs per Center

**Figure 2.**
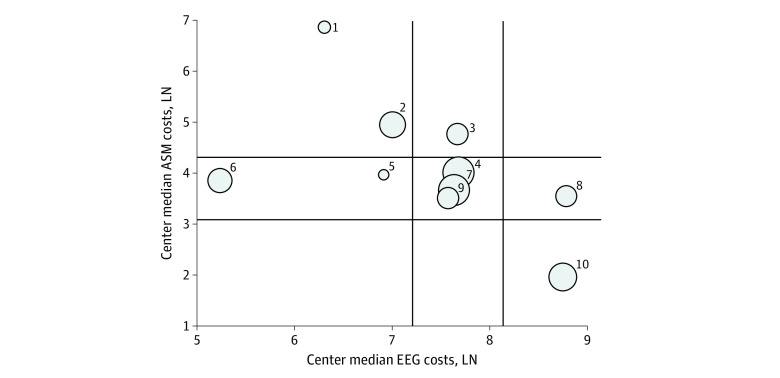
Adjusted Electroenchaphalography (EEG) and Antiseizure Medication (ASM) Costs by Center for Survivors Without Neurodevelopmental Impairment Scatterplot where each bubble represents the volume of hypoxic-ischemic encephalopathy cases in each center and the bubble position represents the center median ASM and EEG costs (expressed on the logarithmic scale). The vertical axis indicates the 25th and 75th percentile of the median overall EEG center costs while the horizontal axis of the median overall ASM center costs. Centers with higher median EEG costs had lower median ASM costs.

When analyzing costs across low-, medium-, and high-cost centers after adjusting for covariates, there was no significant association between hospitalization costs during the first 4 days in high- or medium-cost centers and death or NDI (OR, 1.15 [95% CI, 0.65-2.02] and OR, 0.76 [95% CI, 0.35-1.67], respectively; *P* = .55) (Table 3). High– and medium–EEG cost centers had lower odds of death or NDI compared with low–EEG cost centers (high vs low: OR, 0.30 [95% CI, 0.16-0.57]; medium vs low: OR, 0.29 [95% CI, 0.13-0.62]; *P* < .001) (Table 3). High– (OR, 2.35 [95% CI, 1.19-4.66]) and medium–laboratory cost centers (OR, 1.93 [95% CI, 1.07-3.47]) had higher odds of death or NDI compared with low-cost centers (*P* = .02) (Table 3). Compared with low–ASM cost centers, high–ASM cost centers had higher odds of death (OR, 3.72 [95% CI, 1.51-9.18]) or NDI (OR, 1.56 [95% CI, 0.71-3.42]) (*P* = .007) (Table 3). We did not observe a significant association between EEG and ASM costs (Figure 2).

**Table 3.** Association Between Adjusted Hospitalization and Neonatal Neurocritical Care Cost by Terciles and Death or NDI^a^

Cost category	Odds ratio (95% CI)		*P* value
	Medium vs low tercile	High vs low tercile	
**Per center**			
Hospitalization first 4 d	1.15 (0.65-2.02)	0.76 (0.35-1.66)	.55
EEG	0.30 (0.16-0.57)	0.29 (0.13-0.62)	<.001
Laboratory	2.35 (1.19-4.66)	1.93 (1.07-3.47)	.02
ASM	1.56 (0.71-3.42)	3.72 (1.51-9.18)	.007
**Per case**			
Hospitalization first 4 d	1.47 (0.66-3.29)	1.81 (0.63-5.19)	.51
EEG	0.89 (0.40-1.98)	1.00 (0.35-2.91)	.95
Laboratory	5.22 (2.18-12.48)	4.93 (1.59-15.20)	.001
ASM	1.27 (0.42-3.86)	1.09 (0.31-3.77)	.90

Abbreviations: ASM, antiseizure medication; EEG, electroencephalography; NDI, neurodevelopmental impairment.

^a^ Covariates adjusted for include hypoxic-ischemic encephalopathy severity, EEG, confirmed seizure, inhaled nitric oxide, extracorporeal membrane oxygenation, socioeconomic status, and ventilator days.

### Adjusted Costs per Case

When analyzing costs across low-, medium-, and high-cost cases, there was no significant association between high– (OR, 1.81 [95% CI, 0.63-5.19]) or medium–hospitalization costs (1.47 [95% CI, 0.66-3.29]) and death or NDI (*P* = .51) (Table 3). High– (OR, 4.90 [95% CI, 1.60-15.20]) and medium–laboratory case costs (5.22 [95% CI, 2.18-12.50]) had higher odds of death or NDI compared with low–case cost centers (*P* = .001) (Table 3). EEG and ASM case costs were not significantly associated with the risk for death or NDI (Table 3).

## Discussion

In neonates with HIE treated with TH at regional children’s hospitals, total hospitalization costs during the first 4 days of age were not associated with risk for death or NDI. Meanwhile, infants cared for at high–EEG cost centers had lower odds of death or NDI despite controlling for differences in clinical severity and medical comorbidities. Conversely, we found that higher laboratory costs and ASM costs were not associated with improved survival without NDI.

TH is a cost-effective strategy in the management of neonates with moderate to severe HIE,^1^ however, components of its associated NNCC have not been fully studied. We previously reported the median (IQR) hospitalization cost per case among 19 NICUs participating in CHND for survivors was $58 552 ($32 476-$130 203) and for nonsurvivors was $29 760 ($16 897-$61 399).^2^ Center differences accounted for 29% of variation in total hospitalization cost with the widest cost variability across centers being EEG.^2^ The Canadian Neonatal Network reported practice and outcome variations in neonates with HIE treated with TH across Canada.^9^ Practice differences in this report were mainly in the use of inotropes, iNO, blood products, and feeding during TH. The rates of mortality and brain injury also varied significantly across NICUs. None of these prior reports evaluated whether costs or resource utilization translated into improved neurodevelopmental outcomes.

Variations in costs can occur at the patient and center level, therefore, we ranked costs to account for clinician variation as well as center-specific practices. We did not find hospitalization costs during the first 4 days of age, at center nor case level to be associated with outcomes. We focused on more than 4 days of age as our prior work showed that hospital costs are associated with length of stay.^2^ We have also observed the median age of death in neonates with HIE is 4 days, with the majority occurring after withdrawal of life sustaining support.^20^ Therefore, while using a cutoff of death greater than 4 days excluded 45% of the patients who died, we found that baseline characteristics were similar between deaths within 4 and after 4 days. Additionally, those that died within 4 days had lower costs which would have skewed our cost quantification due to unequal observation period. Although unadjusted hospitalization costs were significantly higher for the death or NDI group, it was reassuring to find that adjusted hospitalization costs were not associated with long-term disease burden.

The association between higher center EEG costs and improved outcomes is an interesting finding that suggests further study may indicate possible practice changes. Preclinical animal studies suggest that seizures are not just a manifestation of brain injury but themselves cause additional brain injury, behavioral deficits, and age-dependent impairment of neurogenesis.^21,22,23,24^ In neonates with HIE, seizures are a manifestation of underlying brain injury and high seizure burden is associated with severity of encephalopathy, more severe brain injury, and worse developmental outcomes in survivors of HIE.^4,25,26,27^ Kharoshayanka et al^25^ noted more than 9-fold higher odds of poor outcome if seizure burden exceeded 40 minutes and an 8-fold increase if hourly seizure burden was greater than 13 minutes per hour. Early detection, identification and treatment of seizures could therefore potentially improve outcomes. However, neonatal seizures are often subclinical and difficult to identify even for experienced clinicians.^19,28^ Continuous EEG monitoring allows earlier detection of electrographic seizures, reduces the time to ASM therapy and decreases seizure burden.^19,28,29,30,31^ A recent study by Hunt et al^32^ in contrast did not reveal such utility or benefit to using aEEG monitoring to direct treatment of electrographic seizures with regards to death or disability at 2 years of age. However, the analyses did not account for differences in ASM use or seizure severity and/or burden.^32^ Although our study showed an association between higher EEG costs and improved outcomes, we were not able to account for aEEG use as the analysis was limited to CTC codes for EEG hospital charges.

EEG monitoring could limit or reduce unnecessary ASM use, for example, by identifying paroxysmal events without electrographic correlate that can be mistaken for seizures. Jan et al^33^ evaluated their single center data during 3 time periods of changing EEG monitoring practices (period 1: single 1 hour EEG when seizure was suspected; period 2: brief EEG plus aEEG for the duration of TH and repeat single EEG during rewarming; period 3: continuous EEG [cEEG] for the duration of TH plus 12 hours during and after rewarming). They found a higher sensitivity and specificity of cEEG for seizure detection, and fewer infants prescribed ASM when undergoing aEEG (38% reduction) and cEEG (67% reduction) compared to single EEG use alone.^33^ This suggests that EEG monitoring, specifically cEEG monitoring, may be helpful to avoid overtreatment. It is reassuring that in our study, center median EEG costs did not correlate with higher ASM costs. It is possible that higher EEG costs (correlated with use) may be associated with lower ASM costs per use, however, this needs to be further investigated. We also found that higher ASM cost was associated with higher odds of death or NDI at the center but not case level, after controlling for HIE severity and presence of EEG seizures. Animal studies have shown that ASMs cause cell death, however, human studies are limited.^34,35^ Our study findings suggest that center-driven practices such as increased EEG monitoring may play a role in improving neurodevelopmental outcomes in neonates with HIE, and are consistent with neuromonitoring guidelines proposed by the American Clinical Neurophysiology Society^36^ and Newborn Brain Society.^37^

It is important to note that higher laboratory costs at the center and case level, indicative of more intensive laboratory testing, were not associated with improved outcomes. These findings are not surprising as laboratory schedules tend to be protocol driven at some centers, whereas at other sites NICU clinicians may individualize laboratory testing based on individual clinical indications. The Neonatal Research Network hypothermia trial left surveillance for organ dysfunction at the discretion of treating physicians and described as “per routine clinical care.”^38^ Although the higher laboratory costs may be reflective of the severity of medical illness which may correlate with risk for adverse neurologic outcomes, our analyses were adjusted for medical comorbidities such as iNO/ECMO and need for mechanical ventilation.

### Limitations

This study has several limitations which include being a registry study and observational in nature, and therefore has bias inherent to retrospective analyses (despite prospectively collected data). We evaluated costs during the first 4 days of age to focus on the period of NNCC during TH and rewarming and to reduce the variability in costs that are related to differences in length of hospital stay. We were interested in the most acute period of care with likely the highest variability in practice to assess whether investment in neurocritical care during this period affects survival and neurodevelopmental outcomes. This approach is consistent with a prior study where illness severity during the first 4 days of age, not just during the initial hypoxic event, was shown to be a predictor of adverse outcomes in neonates with HIE treated with TH.^39^ Neuroimaging costs such as brain MRI were not included in this study given that this is routinely performed after the rewarming period at most centers. Furthermore, brain MRI is a relatively small proportion of overall hospital costs, is not as variable compared with other categories of costs,^2^ and is performed mainly for prognostic rather than interventional purposes. Additionally, our cost analysis was limited to hospital charges coded by PHIS, therefore, this study did not consider costs of aEEG, hours of monitoring, nor account for professional charges that may not be captured in PHIS. We also acknowledge that there may be differences between centers not accounted in this study that can affect patient outcomes.

## Conclusions

Higher resource utilization as measured by hospitalization costs during the first 4 days of age for neonates with HIE treated with TH was not associated with neurodevelopmental outcomes. Infants cared for in centers ranked in the highest tercile for EEG costs had lower odds of death or NDI, whereas higher laboratory costs were not associated with improved outcomes. These findings may serve as the first steps toward identifying aspects of NNCC which relate to neurodevelopmental outcomes and guide future care in this patient population.

## Notes

Supplement 1. **eTable 1.** Entire Cohort Patient Characteristics **eTable 2.** Demographics and Outcomes of Study Population and Excluded Deaths ≤4 Days **eTable 3.** Demographics and Outcomes of Survivors With or Without Developmental Data vs Lost to Follow-up **eTable 4.** NDI Component Rates in Infants Who Survived With NDI **eTable 5.** Unadjusted Costs by Death, Survivors With NDI and Without NDI **eTable 6.** Unadjusted Costs by Death<4 Days, Death >4 Days, Survivors With NDI and Without NDI

Supplement 2.
Nonauthor Collaborators

Supplement 3.
Data Sharing Statement
