# Supplementary material for: Association of Hospital Resource Utilization With Neurodevelopmental Outcomes in Neonates With Hypoxic-Ischemic Encephalopathy
Source: JAMA Netw Open. 2023 Mar 21;6(3):e233770. doi: 10.1001/jamanetworkopen.2023.3770 (PMC10031395; doi:10.1001/jamanetworkopen.2023.3770)
Supplement: Supplement 1. — eTable 1. Entire Cohort Patient Characteristics eTable 2. Demographics and Outcomes of Study Population and Excluded Deaths ≤4 Days eTable 3. Demographics and Outcomes of Survivors With or Without Developmental Data vs Lost to Follow-up eTable 4. NDI Component Rates in Infants Who Survived With NDI eTable 5. Unadjusted Costs by Death, Survivors With NDI and Without NDI eTable 6. Unadjusted Costs by Death<4 Days, Death >4 Days, Survivors With NDI and Without NDI [file jamanetwopen-e233770-s001.pdf]

Supplemental Online Content

Quinones Cardona V, Rao R, Zaniletti I, et al; Children's Hospitals Neonatal Consortium. Association of hospital resource utilization with neurodevelopmental outcomes in neonates with hypoxic-ischemic encephalopathy. *JAMA Netw Open.* 2023;6(3):e233770. doi:10.1001/jamanetworkopen.2023.3770

eTable 1. Entire Cohort Patient Characteristics

eTable 2. Demographics and Outcomes of Study Population and Excluded Deaths ≤4 Days

eTable 3. Demographics and Outcomes of Survivors With or Without Developmental Data vs Lost to Follow-up

eTable 4. NDI Component Rates in Infants Who Survived With NDI

eTable 5. Unadjusted Costs by Death, Survivors With NDI and Without NDI

eTable 6. Unadjusted Costs by Death<4 Days, Death >4 Days, Survivors With NDI and Without NDI

This supplemental material has been provided by the authors to give readers additional information about their work.

© 2023 Quinones Cardona V et al. *JAMA Network Open.*

eTable 1. Entire cohort patient characteristics

Characteristics	Overall n=1693 n (%)	Excluded n=1312 n (%)	Study Population n=381 n (%)	P value
**GA, median [IQR] in weeks**	39 [38,40]	39 [38,40]	39 [38,40]	0.22
**BW, median [IQR] in grams**	3295 [2930,3700]	3290 [2920,3686]	3314 [2950,3788]	0.11
**Female**	712 (42.1)	550 (41.9)	162 (42.5)	0.86
**Maternal Race**				
White	1033 (61.0)	796 (60.7)	237 (62.2)	0.63
Black	364 (21.5)	285 (21.7)	79 (20.7)	0.72
Other	232 (13.7)	174 (13.3)	58 (15.2)	0.35
Unknown	64 (3.8)	57 (4.3)	7 (1.8)	0.022
**Hispanic**	269 (15.9)	206 (15.7)	63 (16.5)	0.88
**Birth location**				
Birthing Center, not hospital	8 (0.5)	6 (0.5)	2 (0.5)	0.99
Home	35 (2.1)	28 (2.1)	7 (1.8)	0.99
Hospital	1650 (97.5)	1278 (97.4)	372 (97.6)	0.99
**Delivery type**				
Vaginal, non-operative	461 (27.2)	354 (27.0)	107 (28.1)	0.69
Vaginal, operative	170 (10.0)	133 (10.1)	37 (9.7)	0.85
Cesarean	1059 (62.6)	822 (62.7)	237 (62.2)	0.90
**APGAR @10 min ≤5**	461 (27.2)	354 (27.0)	107 (28.1)	0.69
**Presenting pH <7**	235 (13.9)	153 (11.7)	82 (21.5)	<0.001
**Cord gas BD (or 1st hr gas), median [IQR]**	7 [6.8,7.2]	7 [6.9,7.1]	7 [6.8,7.2]	0.45
**Perinatal sentinel event**				
Nuchal Cord	312 (18.4)	240 (18.3)	72 (19.0)	0.82
Cord Prolapse	59 (3.5)	44 (3.4)	15 (3.9)	0.63
Uterine Rupture	82 (4.8)	58 (4.4)	24 (6.3)	0.14
Placental Abruption	193 (11.4)	145 (11.1)	48 (12.6)	0.14
Fetal Distress	473 (27.9)	393 (29.9)	80 (21.0)	0.001
**Encephalopathy severity**				
Mild Moderate	247 (14.6)	0 (0)	247 (64.8)	<0.001
Severe	1330 (78.6)	1312 (100)	18 (4.7)	0.06
Unknown	116 (6.9)	0 (0.0)	116 (30.5)	<0.001
**Head cooling**	272 (16.1)	213 (16.2)	59 (15.5)	0.75
**Whole body cooling**	1411 (83.3)	1085 (82.7)	326 (85.6)	0.21
**Clinical seizures**	142 (8.4)	91 (6.9)	51 (13.4)	<0.001
**EEG/aEEG confirmed seizures**	369 (21.8)	264 (20.1)	105 (27.6)	0.002
**No seizures**	1073 (63.4)	871 (66.4)	202 (53.0)	<0.001
**ECMO**	56 (3.3)	44 (3.4)	12 (3.2)	0.99
**iNO**	163 (9.6)	136 (10.4)	27 (7.1)	0.18
**Tracheostomy**	7 (0.4)	5 (0.4)	2 (0.5)	0.66
**Gastrostomy tube**	81 (4.8)	61 (4.7)	20 (5.3)	0.68
**Ventilator days, median [IQR]**	2 [0,6]	2 [0,5]	4 [0,7]	<0.001
**Length of stay, median [IQR]**	12 [8,19]	11 [8,19]	12 [8,20]	0.15

aEEG *amplitude integrated electroencephalogram*, BD *base deficit*, BW *birthweight*, EEG *electroencephalogram*, ECMO *extracorporeal membrane oxygenation*, GA *gestational age*, iNO *inhaled nitric oxide*, IQR *interquartile range*, NDI *neurodevelopmental impairment*
p<0.05 considered statistically significant

© 2023 Quinones Cardona V et al. *JAMA Network Open.*

eTable 2. Demographics and Outcomes of study population and excluded deaths ≤4 days

Characteristics	Overall n=446 n (%)	Death ≤4 days n=65 n (%)	Death>4 days n=80 n (%)	Survivors with NDI n=64 n (%)	Survivors without NDI n=237 n (%)	P value
**GA, median [IQR] in weeks**	39 [38,40]	39 [38,49]	39 [38,40]	39 [38,40]	39 [38,40]	0.69
**BW, median [IQR] in grams**	3294 [2950,3750]	3220 [2975, 3640]	3337 [2915,3840]	3378 [3000,3780.5]	3280 [2960,3751]	0.46
**Female**	194 (43.5)	32 (49.2)	31 (38.8)	31 (48.4)	100 (42.2)	0.49
**Maternal Race**						
White	277 (62.1)	40 (61.5)	43 (53.8)	41 (64.1)	153 (64.6)	0.38
Black	90 (20.2)	11 (16.9)	21 (26.3)	13 (20.3)	45 (19.0)	0.49
Other	58 (15.2)	10 (15.4)	14 (17.5)	7 (10.9)	37 (15.6)	0.75
Unknown	11 (2.5)	4 (6.2)	2 (2.5)	3 (4.7)	2 (0.8)	0.02
**Hispanic**, n (%)	67 (15)	4 (6.2)	14 (17.5)	10 (15.6)	39 (16.5)	0.23
**Birth location**						
Birthing Center, not hospital	2 (0.5)	0 (0)	1 (1.3)	1 (1.6)	0 (0.0)	0.5
Home	9 (2.02)	2 (3.08)	2 (2.5)	0 (0.0)	5 (2.1)	0.75
Hospital	435 (97.5)	63 (96.9)	77 (96.3)	63 (98.4)	232 (97.9)	0.75
**Delivery type**						
Vaginal, non-operative	121 (27.1)	14 (21.5)	16 (20.0)	19 (29.7)	72 (30.4)	0.21
Vaginal, operative	45 (10.1)	8 (12.3)	9 (11.3)	8 (12.5)	20 (8.4)	0.58
Cesarean	280 (62.8)	43 (66.2)	55 (68.7)	37 (57.8)	145 (61.2)	0.49
**APGAR @10 min ≤5**	234 (52.5)	46 (70.8)	55 (68.8)	39 (60.9)	94 (39.7)	<0.001
**Presenting pH <7**	97 (21.7)	15 (23.1)	11 (13.8)	21 (32.8)	50 (21.1)	0.05
**Cord gas BD (or 1st hr gas), median [IQR]**	17 [11,22]	23 [13,27]	18 [8,22]	18.5 [15,24]	15 [10,20]	0.02
**Perinatal sentinel event**						
Nuchal Cord	75 (16.8)	3 (4.6)	16 (20.0)	13 (20.3)	43 (18.1)	0.02
Cord Prolapse	16 (3.6)	1 (1.5)	2 (2.5)	4 (6.3)	9 (3.8)	0.53
Uterine Rupture	29 (6.5)	5 (7.7)	7 (8.8)	7 (10.9)	10 (4.2)	0.14
Placental Abruption	55 (12.3)	7 (10.8)	7 (8.8)	11 (17.2)	30 (12.7)	0.14
Fetal Distress	100 (22.4)	20 (30.8)	20 (25.0)	19 (29.7)	41 (17.3)	0.034
**Encephalopathy severity**						
Mild Moderate	253 (56.7)	6 (9.2)	5 (6.3)	47 (73.4)	195 (82.3)	<.0001
Severe	175 (39.2)	59 (90.8)	70 (87.5)	1 (1.6)	12 (5.1)	<0.001
Unknown	18 (4.1)		5 (6.3)	16 (25.0)	30 (12.7)	0.131
**Head cooling**	62 (13.9) +	3 (4.6)	12 (15.0)	11 (17.2)	36 (15.2)	0.09
**Whole body cooling**	397 (87.8) +	61 (93.8)	68 (85.0)	57 (89.1)	201 (84.8)	0.24
**Clinical seizures**	58 (13)	7 (10.8)	15 (18.8)	4 (6.3)	32 (13.5)	0.16
**EEG/aEEG confirmed seizures**	125 (28.0)	20 (30.8)	36 (45.0)	21 (32.8)	48 (20.3)	<0.001
**No seizures**	238 (53.4)	36 (55.4)	21 (26.3)	36 (56.3)	145 (61.2)	<0.001
**ECMO**	14 (3.1)	2 (3.1)	5 (6.3)	1 (1.6)	6 (2.5)	0.37
**iNO**	94 (21.1)	20 (30.8)	26 (32.5)	14 (21.9)	34 (14.3)	0.005
**Tracheostomy**	2 (0.5)	0 (0)	0 (0)	0 (0)	2 (0.8)	0.99
**Gastrostomy tube**	20 (5.3)	0 (0)	0 (0)	9 (14.1)	11 (4.6)	<0.001
**Ventilator days, median [IQR]**	4 [0,7]	2 [1,3]	8 [6,11]	4 [2,8]	4 [1,7]	<0.001
**Length of stay, median [IQR]**	11 [6,18]	1 [1,2]	7 [5,11]	17 [9, 29]	13 [9,21]	<0.001

aEEG *amplitude integrated electroencephalogram*, BD *base deficit*, BW *birthweight*, EEG *electroencephalogram*, ECMO *extracorporeal membrane oxygenation*, GA *gestational age*, iNO *inhaled nitric oxide*, IQR *interquartile range*, NDI *neurodevelopmental impairment*
p<0.05 considered statistically significant

© 2023 Quinones Cardona V et al. *JAMA Network Open.*

eTable 3. Demographics and Outcomes of Survivors with or without developmental data vs lost to follow-up

Characteristics	Overall<br>n=1513<br>n (%)	Survivors with or without developmental data<br>n=301<br>n (%)	Lost to follow up<br>n=1212<br>n (%)	P value
**GA, median [IQR] in weeks**	39 [38,40]	39 [38,40]	39 [38,40]	0.38
**BW, median [IQR] in grams**	3300 [2925,3710]	3300 [2913.5,3690]	3310 [2970,3751]	0.18
**Female**	634 (41.9)	503 (41.5)	131 (43.5)	0.56
**Maternal Race**				
White	923 (61)	729 (60.1)	194 (64.4)	0.19
Black	330 (21.8)	272 (22.4)	58 (19.3)	0.24
Other	205 (13.5)	161 (13.3)	44 (14.6)	0.57
Unknown	55 (3.6)	50 (4.1)	5 (1.7)	0.04
**Hispanic**	243 (16.1)	194 (16.0)	49 (16.3)	0.93
**Birth location**				
Birthing Center, not hospital	7 (0.5)	6 (0.5)	1 (0.3)	0.99
Home	30 (1.9)	25 (2.1)	5 (1.7)	0.99
Hospital	1476 (97.5)	1181 (97.4)	295 (98.0)	0.99
**Delivery type**				
Vaginal, non-operative	427 (28.2)	336 (27.7)	91 (30.2)	0.39
Vaginal, operative	150 (9.9)	122 (10.1)	28 (9.3)	0.75
Cesarean	933 (61.7)	751 (61.9)	182 (60.5)	0.64
**APGAR @10 min ≤5**	669 (44.2)	536 (44.2)	133 (44.2)	0.99
**Presenting pH <7**	203 (13.4)	132 (10.9)	71 (23.6)	<0.001
**Cord gas BD (or 1st hr gas), median [IQR]**	15.6 [11,19]	15.1 [11,19]	16 [12,21]	0.37
**Perinatal sentinel event**				
Nuchal Cord	289 (19.1)	233 (19.2)	56 (18.6)	0.87
Cord Prolapse	55 (3.6)	42 (3.5)	13 (4.3)	0.49
Uterine Rupture	67 (4.4)	50 (4.1)	17 (5.7)	0.27
Placental Abruption	169 (11.2)	128 (10.6)	41 (13.6)	0.27
Fetal Distress	421 (27.8)	361 (29.8)	60 (19.9)	0.001
**Encephalopathy severity**				
Mild Moderate	1195 (78.9)	953 (78.6)	242 (80.4)	0.53
Severe	207 (13.7)	161 (13.3)	46 (15.3)	0.53
Unknown	111 (7.3)	98 (8.1)	13 (4.3)	0.08
**Head cooling**	240 (15.9)	193 (15.9)	47 (15.6)	0.93
**Whole body cooling**	1264 (83.5)	1006 (83)	258 (85.7)	0.29
**Clinical seizures**	114 (7.5)	78 (6.4)	36 (11.9)	0.002
**EEG/aEEG confirmed seizures**	297 (19.6)	228 (18.8)	69 (22.9)	0.12
**No seizures**	1009 (66.7)	828 (68.3)	181 (60.1)	0.008
**ECMO**	46 (3.0)	39 (3.2)	7 (2.3)	0.57
**iNO**	247 (16.3)	199 (16.4)	48 (15.9)	0.79
**Tracheostomy**	1513 (100)	1212 (100)	301 (100)	0.50
**Gastrostomy tube**	1513 (100)	1212 (100)	301 (100)	0.99
**Ventilator days, median [IQR]**	5 [2,8]	5 [2,8]	4 [2,7]	0.29
**Length of stay, median [IQR]**	12 [9,20]	12 [9,20]	13 [9,22]	0.01

aEEG *amplitude integrated electroencephalogram*, BD *base deficit*, BW *birthweight*, EEG *electroencephalogram*, ECMO *extracorporeal membrane oxygenation*, GA *gestational age*, iNO *inhaled nitric oxide*, IQR *interquartile range*, NDI *neurodevelopmental impairment*
p<0.05 considered statistically significant

© 2023 Quinones Cardona V et al. *JAMA Network Open.*

eTable 4. NDI component rates in infants who survived with NDI

Component	Frequency (%) n=64
Composite cognitive score <85	27 (42.2)
Composite motor score <70	15 (23.4)
Deafness	1 (1.6)
Blindness	0 (0)
GFMCS score≥2	11 (17.2)

eTable 5. Unadjusted Costs by death, survivors with NDI and without NDI

Costs	**Total** Median $ [IQR] n=381	**Death >4 days** Median $ [IQR] n=80	**Survivors with NDI** Median $ [IQR] n=64	**Survivors without NDI** Median $ [IQR] n=237	**P value**
**Total hospitalization**	26,420 [21,664, 36,610]	33,033 [24,993, 41,144]	24,777 [22,419, 35,694]	25,285 [20,964, 34,030]	<0.001
**EEG**	2,417 [1,293, 4,567]	2,303 [1,264, 4,715]	1,615 [994, 3,004]	2,454 [1,462, 4,644]	0.09
**Laboratory**	3,695 [1,984, 5,425]	5498 [4,224, 8,347]	3,857 [2,504, 4,807]	2,707 [1,570, 4,698]	<0.001
**Anti-seizure medications**	40 [17, 100]	41 [15, 149]	67 [32,121]	34 [16, 81]	0.05

eTable 6. Unadjusted costs by death<4 days, death >4 days, survivors with NDI and without NDI

**Costs**	**Total** Median $ [IQR] n=381	**Death ≤4 days** Median $ [IQR] n=65	**Death >4 days** Median $ [IQR] n=80	**Survivors with NDI** Median $ [IQR] n=64	**Survivors without NDI** Median $ [IQR] n=237	**P value**
**Total hospitalization**	26,420 [21,664, 36,610]	15,766 [8,216, 23,204]	33,033 [24,993, 41,144]	24,777 [22,419, 35,694]	25,285 [20,964, 34,030]	<0.001
**EEG**	2,417 [1,293, 4,567]	677 [450, 1,023]	2,303 [1,264, 4,715]	1,615 [994, 3,004]	2,454 [1,462, 4,644]	<0.001
**Laboratory**	3,695 [1,984, 5,425]	2,980 [1,594, 4,529]	5498 [4,224, 8,347]	3,857 [2,504, 4,807]	2,707 [1,570, 4,698]	<0.001
**Anti-seizure medications**	40 [17, 100]	46 [12.3, 100]	41 [15, 149]	67 [32,121]	34 [16, 81]	0.09

© 2023 Quinones Cardona V et al. *JAMA Network Open.*
