# Supplementary material for: Association of Hospital Resource Utilization With Neurodevelopmental Outcomes in Neonates With Hypoxic-Ischemic Encephalopathy
Source: JAMA Netw Open. 2023 Mar 21;6(3):e233770. doi: 10.1001/jamanetworkopen.2023.3770 (PMC10031395; doi:10.1001/jamanetworkopen.2023.3770)
Supplement: Supplement 2. — Nonauthor Collaborators [file jamanetwopen-e233770-s002.pdf]

Supplemental Online Content: Nonauthor Collaborators
*First name, last name, and suffix (if applicable) are required and will appear in PubMed.

***Group Name(s): Children's Hospitals Neonatal Consortium (CHNC)**							
***First Name and Middle Initial(s)**	***Last Name**	***Suffix (eg, Jr, III)**	Academic Degrees	Institution	Location (city, state/province, country)	Role or Contribution, eg, chair, principal investigator	Group (if more than 1 Group listed in the byline) and/or Subgroup (eg, Steering Committee)
Nathalie	Maitre		MD, PhD	Children's Healthcare of Atlanta at Scottish Rite	Atlanta, GA, USA	Data collection	Children's Hospitals Neonatal Consortium (CHNC)
Amit	Mathur		MD	St. Louis Children's Hospital (past)	Saint Louis, MO, USA	Data collection, Past Site Sponsor	Children's Hospitals Neonatal Consortium
Eugenia	Pallotto		MD, MSCE	Levine Children's Hospital	Charlotte, NC,USA	Site Sponsor, Data collection	Children's Hospitals Neonatal Consortium (CHNC)
Danielle	Smith		MD	Colorado Children's Hospital	Denver, CO, USA	Data collection	Children's Hospitals Neonatal Consortium (CHNC)
Mark	Speziale		MD	Rady Children's Hospital	San Diego, CA, USA	Data collection	Children's Hospitals Neonatal Consortium (CHNC)
Toby	Yanowitz		MD	Children's Hospital of Pittsburgh of UPMC	Pittsburg, PA, USA	Past member, Data collection	Children's Hospitals Neonatal Consortium (CHNC)
Beverly	Brozanski		MD, CPHQ	St. Louis Children's Hospital	St Louis, MO, USA	Executive Committee	Children's Hospitals Neonatal Consortium (CHNC)
Jacquelyn	Evans		MD	Children's Hospital of Philadelphia	Philadelphia, PA , USA	Executive Committee	Children's Hospitals Neonatal Consortium (CHNC)
Theresa	Grover		MD, MAS	Children's Hospital Colorado	Denver, CO, USA	Executive Committee	Children's Hospitals Neonatal Consortium (CHNC)
Karna	Murthy		MD, MSc	Ann & Robert H. Lurie Children's Hospital of Chicago	Chicago, IL, USA	Executive Committee	Children's Hospitals Neonatal Consortium (CHNC)

Supplemental Online Content: Nonauthor Collaborators
*First name, last name, and suffix (if applicable) are required and will appear in PubMed.

***First Name and Middle Initial(s)**	***Last Name**	***Suffix (eg, Jr, III)**	Academic Degrees	Institution	Location (city, state/province, country)	Role or Contribution, eg, chair, principal investigator	Group (if more than 1 Group listed in the byline) and/or Subgroup (eg, Steering Committee)
Michael	Padula		MD, MBI	Children’s Hospital of Philadelphia	Philadelphia, PA , USA	Executive Committee	Children's Hospitals Neonatal Consortium (CHNC)
Anthony	Piazza		MD	Children’s Healthcare of Atlanta at Egleston	Atlanta, GA, USA	Executive Committee	Children's Hospitals Neonatal Consortium (CHNC)
Kristina	Reber		MD	Nationwide Children’s Hospital	Columbus, OH, USA	Executive Committee	Children's Hospitals Neonatal Consortium (CHNC)
Billie	Short		MD	Children’s National Medical Center	Washington, DC, USA	Executive Committee	Children's Hospitals Neonatal Consortium (CHNC)
David	Durand		MD	Children's Hospital & Research Center Oakland (past)	Oakland, CA, USA	Past member	Children's Hospitals Neonatal Consortium (CHNC)
Francine	Dykes		MD	Children's Hospital of Atlanta (past)	Atlanta, GA, USA	Past member	Children's Hospitals Neonatal Consortium (CHNC)
Jeanette	Asselin		MS, RRT-NPS	Children's Hospital & Research Center Oakland (past)	Oakland, CA, USA	Past member	Children's Hospitals Neonatal Consortium (CHNC)
Kevin	Sullivan		MD	Alfred I. duPont Hospital for Children	Wilmington, DE, USA	Site Sponsor	Children's Hospitals Neonatal Consortium (CHNC)
Victor	McKay		MD	All Children's Hospital Johns Hopkins Medicine	St. Petersburg, FL, USA	Site Sponsor	Children's Hospitals Neonatal Consortium (CHNC)
Jamie	Limjoco		MD	American Family Hospital	Madison, WI, USA	Site Sponsor	Children's Hospitals Neonatal Consortium (CHNC)

Supplemental Online Content: Nonauthor Collaborators

*First name, last name, and suffix (if applicable) are required and will appear in PubMed.

***First Name and Middle Initial(s)**	***Last Name**	***Suffix (eg, Jr, III)**	Academic Degrees	Institution	Location (city, state/province, country)	Role or Contribution, eg, chair, principal investigator	Group (if more than 1 Group listed in the byline) and/or Subgroup (eg, Steering Committee)
Lori	Haack		SCT	American Family Hospital	Madison, WI, USA	Site Sponsor	Children's Hospitals Neonatal Consortium (CHNC)
Narenda	Dereddy		MD	Advent Health for Children	Orlando, FL, USA	Site Sponsor	Children's Hospitals Neonatal Consortium (CHNC)
Raj	Wadhawan		MD	Advent Health for Children	Orlando, FL, USA	Site Sponsor	Children's Hospitals Neonatal Consortium (CHNC)
Karna	Murthy		MD	Ann & Robert H. Lurie Children's Hospital of Chicago	Chicago, IL, USA	Site Sponsor	Children's Hospitals Neonatal Consortium (CHNC)
Gustave	Falciglia		MD	Ann & Robert H. Lurie Children's Hospital of Chicago	Chicago, IL, USA	Site Sponsor	Children's Hospitals Neonatal Consortium (CHNC)
Becky	Rogers		MD	Arkansas Children's Hospital		Site Sponsor	Children's Hospitals Neonatal Consortium (CHNC)
Anne	Hansen		MD	Boston Children's Hospital	Boston, MA, USA	Site Sponsor	Children's Hospitals Neonatal Consortium (CHNC)
Cherrie	Welch		MD	Brenner Children's Hospital	Winston-Salem, NC, USA	Site Sponsor	Children's Hospitals Neonatal Consortium (CHNC)
Beth	Haberman		MD	Cincinnati Children's Hospital	Cincinnati, OH, USA	Site Sponsor	Children's Hospitals Neonatal Consortium (CHNC)
Anthony	Piazza		MD	Children's Healthcare of Atlanta at Egleston	Atlanta, GA, USA	Site Sponsor	Children's Hospitals Neonatal Consortium (CHNC)

Supplemental Online Content: Nonauthor Collaborators
*First name, last name, and suffix (if applicable) are required and will appear in PubMed.

***First Name and Middle Initial(s)**	***Last Name**	***Suffix (eg, Jr, III)**	Academic Degrees	Institution	Location (city, state/province, country)	Role or Contribution, eg, chair, principal investigator	Group (if more than 1 Group listed in the byline) and/or Subgroup (eg, Steering Committee)
Gregory	Sysyn		MD	Children's Healthcare of Atlanta at Scottish Rite	Atlanta, GA, USA	Site Sponsor	Children's Hospitals Neonatal Consortium (CHNC)
Nicole	Birge		MD	Children's Hospital and Medical Center	Omaha, NE, USA	Site Sponsor	Children's Hospitals Neonatal Consortium (CHNC)
THeresa	Grover		MD	Children's Hospital Colorado	Denver, CO, USA	Site Sponsor	Children's Hospitals Neonatal Consortium (CHNC)
Michel	Mikhael		MD	Children's Hospital of Orange County	Orange, CA, USA	Site Sponsor	Children's Hospitals Neonatal Consortium (CHNC)
Irfan	Ahmad		MD	Children's Hospital of Orange County	Orange, CA, USA	Site Sponsor	Children's Hospitals Neonatal Consortium (CHNC)
Michael	Padula		MD	Children's Hospital of Philadelphia	Philadelphia, PA , USA	Site Sponsor	Children's Hospitals Neonatal Consortium (CHNC)
David	Munson		MD	Children's Hospital of Philadelphia	Philadelphia, PA , USA	Site Sponsor	Children's Hospitals Neonatal Consortium (CHNC)
Toby	Yanowitz		MD	Children's Hospital of Pittsburgh of UPMC	Pittsburg, PA, USA	Site Sponsor	Children's Hospitals Neonatal Consortium (CHNC)
Michael	Uhing		MD	Children's Hospital of Wisconsin	Milwaukee, WI, USA	Site Sponsor	Children's Hospitals Neonatal Consortium (CHNC)
Ankur	Datta		MD	Children's Hospital of Wisconsin	Milwaukee, WI, USA	Site Sponsor	Children's Hospitals Neonatal Consortium (CHNC)

Supplemental Online Content: Nonauthor Collaborators
*First name, last name, and suffix (if applicable) are required and will appear in PubMed.

***First Name and Middle Initial(s)**	***Last Name**	***Suffix (eg, Jr, III)**	Academic Degrees	Institution	Location (city, state/province, country)	Role or Contribution, eg, chair, principal investigator	Group (if more than 1 Group listed in the byline) and/or Subgroup (eg, Steering Committee)
Rashmin	Savani		MD	Children’s Medical Center Dallas	Dallas, TX, USA	Site Sponsor	Children's Hospitals Neonatal Consortium (CHNC)
Luc	Brion		MD	Children’s Medical Center Dallas	Dallas, TX, USA	Site Sponsor	Children's Hospitals Neonatal Consortium (CHNC)
Julie	Weiner		DO	Children’s Mercy Hospitals and Clinics	Kansas City, MO, USA	Site Sponsor	Children's Hospitals Neonatal Consortium (CHNC)
Billie	Short		MD	Children’s National Medical Center	Washington, DC, USA	Site Sponsor	Children's Hospitals Neonatal Consortium (CHNC)
Lamia	Soghier		MD	Children’s National Medical Center	Washington, DC, USA	Site Sponsor	Children's Hospitals Neonatal Consortium (CHNC)
Carl	Coghill		MD	Children’s of Alabama	Birmingham, AL, USA	Site Sponsor	Children's Hospitals Neonatal Consortium (CHNC)
Allison	Black		MD	Children’s of Alabama	Birmingham, AL, USA	Site Sponsor	Children's Hospitals Neonatal Consortium (CHNC)
Steven	Chin		MD	Children's Hospital of Los Angeles	Los Angeles, CA, USA	Site Sponsor	Children's Hospitals Neonatal Consortium (CHNC)
Rachel	Chapman		MD	Children's Hospital of Los Angeles	Los Angeles, CA, USA	Site Sponsor	Children's Hospitals Neonatal Consortium (CHNC)
AnneMarie	Golioto		MD	Connecticut Children's Medical Center	Hartford, CT, USA	Site Sponsor	Children's Hospitals Neonatal Consortium (CHNC)

Supplemental Online Content: Nonauthor Collaborators
*First name, last name, and suffix (if applicable) are required and will appear in PubMed.

***First Name and Middle Initial(s)**	***Last Name**	***Suffix (eg, Jr, III)**	Academic Degrees	Institution	Location (city, state/province, country)	Role or Contribution, eg, chair, principal investigator	Group (if more than 1 Group listed in the byline) and/or Subgroup (eg, Steering Committee)
Jonathan	Nedrelow		MD	Cook Children's Medical Center	Fort Worth, TX, USA	Site Sponsor	Children's Hospitals Neonatal Consortium (CHNC)
Annie	Chi		MD	Cook Children's Medical Center	Fort Worth, TX, USA	Site Sponsor	Children's Hospitals Neonatal Consortium (CHNC)
Yvette	Johnson		MD	Cook Children's Medical Center	Fort Worth, TX, USA	Site Sponsor	Children's Hospitals Neonatal Consortium (CHNC)
Mark	Weems		MD	Le Bonheur Children's Hospital	Memphis, TN, USA	Site Sponsor	Children's Hospitals Neonatal Consortium (CHNC)
Kristina	Reber		MD	Nationwide Children's Hospital	Columbus, OH, USA	Site Sponsor	Children's Hospitals Neonatal Consortium (CHNC)
Aaron	Weiss		MD	Nemours Children's Hospital	Orlando, FL, USA	Site Sponsor	Children's Hospitals Neonatal Consortium (CHNC)
Trent	Tripple		MD	Oklahoma Children's Hospital	Oklahoma City, OK, USA	Site Sponsor	Children's Hospitals Neonatal Consortium (CHNC)
Con Yee	Ling		MD	Primary Children's Hospital	Salt Lake City, UT, USA	Site Sponsor	Children's Hospitals Neonatal Consortium (CHNC)
Shrena	Patel		MD	Primary Children's Hospital	Salt Lake City, UT, USA	Site Sponsor	Children's Hospitals Neonatal Consortium (CHNC)
Mark	Speziale		MD	Rady Children's Hospital	San Diego, CA, USA	Site Sponsor	Children's Hospitals Neonatal Consortium (CHNC)

Supplemental Online Content: Nonauthor Collaborators
*First name, last name, and suffix (if applicable) are required and will appear in PubMed.

***First Name and Middle Initial(s)**	***Last Name**	***Suffix (eg, Jr, III)**	Academic Degrees	Institution	Location (city, state/province, country)	Role or Contribution, eg, chair, principal investigator	Group (if more than 1 Group listed in the byline) and/or Subgroup (eg, Steering Committee)
Brian	Lane		MD	Rady Children’s Hospital	San Diego, CA, USA	Site Sponsor	Children's Hospitals Neonatal Consortium (CHNC)
Laurel	Moyer		MD	Rady Children’s Hospital	San Diego, CA, USA	Site Sponsor	Children's Hospitals Neonatal Consortium (CHNC)
William	Engle		MD	Riley Children’s	Indianapolis, IN, USA	Site Sponsor	Children's Hospitals Neonatal Consortium (CHNC)
Lora	Simpson		MD	Riley Children’s	Indianapolis, IN, USA	Site Sponsor	Children's Hospitals Neonatal Consortium (CHNC)
Gregory	Sokol		MD	Riley Children’s	Indianapolis, IN, USA	Site Sponsor	Children's Hospitals Neonatal Consortium (CHNC)
Elizabeth	Jacobsen-Misbe		MD	Seattle Children’s Hospital	Seattle, WA, USA	Site Sponsor	Children's Hospitals Neonatal Consortium (CHNC)
Julie	Lindower		MD	Stead Family Children's Hospital	Iowa City, IO, USA	Site Sponsor	Children's Hospitals Neonatal Consortium (CHNC)
Gautham	Suresh		MD	Texas Children’s Hospital, Houston	Houston, TX, USA	Site Sponsor	Children's Hospitals Neonatal Consortium (CHNC)
Lakshmi	Khatakam		MD	Texas Children’s Hospital, Houston	Houston, TX, USA	Site Sponsor	Children's Hospitals Neonatal Consortium (CHNC)
Art	D'Harlingue		MD	UCSF Benioff Children’s Hospital Oakland	Oakland, CA, USA	Site Sponsor	Children's Hospitals Neonatal Consortium (CHNC)
