# Supplementary material for: Association of Hospital Resource Utilization With Neurodevelopmental Outcomes in Neonates With Hypoxic-Ischemic Encephalopathy
Source: JAMA Netw Open. 2023 Mar 21;6(3):e233770. doi: 10.1001/jamanetworkopen.2023.3770 (PMC10031395; doi:10.1001/jamanetworkopen.2023.3770)
Supplement: Supplement 3. — Data Sharing Statement [file jamanetwopen-e233770-s003.pdf]

Data Sharing Statement

Quinones Cardona. Association of Hospital Resource Utilization With Neurodevelopmental Outcomes in Neonates With Hypoxic-Ischemic Encephalopathy. *JAMA Netw Open*. Published March 21, 2023. doi:10.1001/jamanetworkopen.2023.3770

Data
Data available: No
